# Medicine a Science—Education—The Correlation of Diseases

**Published:** 1907-11

**Authors:** Allen A. Jones

**Affiliations:** Adjunct Professor of Medicine at the University of Buffalo


### BUFFALO MEDICAL JOURNAL.

VOL. LXIII. NOVEMBER, 1907. No. 4

### ORIGINAL COMMUNICATIONS.

### Medicine a Science—Education—The Correlation of Diseases.

**Address delivered at the opening of the Sixty-second Annual session of the Medical Department of the University of Buffalo.**

BY ALLEN A. JONES, M. D.
Adjunct Professor of Medicine at the University of Buffalo.

AN address to young men and women entering upon the study of medicine, and to other young men and women who are pursuing the study of medicine, may well embrace a short discussion of several points of interest.

MEDICINE AS A SCIENCE.

At the outset comes the question of medicine as a science. The mistake is often made of looking upon medicine as a business that one may be apprenticed to and learn as a mechanic learns to do certain things, with his hands and a machine, and accomplish certain results. But medicine should be viewed first as altogether a science, a profoundly deep science, a science first, last and all the time. It should be considered as a science in its infancy with larger hopes and accomplishments in view for the future. Not only allied to medicine, but a part of it, are the difficult, broad and great sciences of chemistry, anatomy, comparative anatomy, physiology and physiological chemistry, pathology and comparative pathology; they cannot for one moment be divorced from medicine; they must be mastered now and in the future and for all time. There can be no possible question as to the value of biology, botany, physics, psychology, criminology, the languages, logic and metaphysics by one undertaking the mastery of the science of medicine. Education of the deepest, best and highest kind ought fervently to be espoused by one who enters this tremendously broad field. He who enters here without adequate preparation, without a deep and abiding spirit of science, will know keen and bitter disappointment, or ought to know it, when he finds that he is desperately or irremediably handicapped in the severest and hardest of all races. There is a vast difference between the individual who with iron determination holds fast to the sciences of medicine, and the one who further and further relaxes his grip upon our only hope of attaining Truth, until he is no longer scientific, but merely mechanically skilful and artful.

It is not for many to rise higher and higher in scientific attainments as life spins out, but to those occupying even useful, laudable, but lower planes, there is an abiding consciousness that their fellows who, at great personal sacrifice and with unswerving fidelity to ideals, are loyal to science, occupy a higher position and command their admiration and envy. No longer is a physician's success or reputation based upon the size of his practice or of his following, but upon his scientific attainments, interests and sympathies. When a physician inquires with honest thought who of his fellows is greatest, his answer comes promptly—he who strives for greater scientific attainment and truth. I speak at the outset in this vein because I know so well the various and many ways in which your lives as practising physicians will militate against progressive scientific work, but there are many instances of busy physicians and surgeons accomplishing splendid work in special scientific fields and those who hold a faithful desire to continue in special work need feel no discouragement, provided they are prepared to make great personal sacrifice of time, work, and money. Either the time or the money must be given to maintain any degree of scientific research in a medical or surgical practice. When the demands of practice grow too numerous and taxing to longer allow you time and strength for clinical laboratory work, be early to engage younger members of the profession in your chemical, microscopic and special clinical research department, even though the cost be at first hard to bear.

### THE CHOICE OF A LIBRARY.

In gathering together works that will eventually constitute your private library, large or small, it is wise to exercise considerable care in the choice of books. A physician's library should indicate that its possessor endeavors to be scientific and is a scholar, a literateur and a gentleman. While the best books upon the line of work he is interested in may predominate upon the shelves, the collection should contain works of wider and more varied interest. For many years it has been the custom to publish large and many volumed systems of medicine and surgery, and while these constitute some of the most valuable material of a library, it is well for the student and young practitioner to buy but few such, if any, unless the buyer has ample independent means, because a system is always somewhat expensive and, unfortunately, becomes out of date in a surprisingly short time. A student is apt to be perplexed by and to glean little from a system, but when he is well established, the best systems will serve to round out his library and will be found to contain invaluable, well-arranged information upon many vital topics. It is possible, in some instances, to obtain single volumes of a system bearing upon the subject of special interest to the physician.

Finally, it should be impressed upon you that the standard medical and surgical journals should be early subscribed to and regularly read carefully with such despatch as your time will allow. In your earlier years of practice it will be possible for you to keep well abreast of the current medical literature, but in later years many of you will read the journals with greater difficulty because of fatigue and time taxation; but you should strive in the beginning to sift the good grain from the chaff, in order that in later years you may be enabled to gather quickly what you most need from the journals with the greatest economy of time and effort.

### METHODS OF STUDY.

For those of my hearers who are beginners and who are, possibly, not favored by nature with as well ordered minds as their more fortunate fellows, it may not be out of place to dwell for a few minutes upon methods of study. Many students start wrong and are not aware of it. They work hard and attentively but do not progress. Discouragement comes and matters are worse than before. By the inscrutable ordering of that which we call chance, a companion student offers assistance and the two work together. In a short time the one in difficulty begins to experience and manifest a better understanding, a clearer insight, a greater grasp of the subject, the mists gradually sweep away, and pleasure, profit and good standing grow apace. This is explained by the fact that the first student's method of study was fundamentally faulty. He continually went over points and facts without mastering them, without thoroughly understanding them. He went over the ground without taking a fort, as it were.

The lecture or the conference was apparently clear and interesting, but in the quiet of his room the student realized that he could not define one statement that was presented; he failed to correlate the facts one to the other. His good fellow student spent a whole evening working with him upon one definition, mastering it in all its bearings until it became incorporated as knowledge, as a crystallized fact in the understanding, and the next day when the subject came up, his difficulty was lessened. I pray you know now, and through all your student life, that it is useless and wrong for you to skim over facts without mastering them and making them yours. It will be found much more profitable to spend hours and days upon one little, but important, fact until it is indelibly yours, than to allow the exigencies of the course, or the student body, or laziness, or indifference, or diffidence, or any other influence, to hurry you past that fact. It will surely prove a stumbling block afterward. The numerous things you must learn may sometimes prove confusing and disheartening, but you may be encouraged by the assurance that if you will slowly and patiently incorporate one point after another as your very own, each subsequent day's work is made easier and unconsciously you will become possessors of great knowledge. Strive hard not to allow the baneful spirit of the age,—hurry,—to make you superficial. Better a few things known well than many known but superficially.

All persons are not constituted alike, and in early school days an observing teacher should discover that one pupil takes up a special subject with pleasure and ease, while another subject offers him serious difficulty. So, in the medical course, it is a common occurrence to find a student going along well with some topics and poorly or badly with others; possibly only one or two. Those of you who find one subject a stumbling block I advise, if possible, to engage the assistance and coöperation of a fellow-worker who masters that subject with a different and a better method than you adopt.

Before leaving this point it may be well to caution some of you that, the contrary doctrine notwithstanding, for you at least the condition of your brain matter has much to do with your mental efficiency; and from the utilitarian standpoint, if from no other, all habits of life having a detrimental physical effect will surely lessen your mental powers. Exercise in the fresh air, sufficient rest and recreation, and sane living should be regularly observed.

### CHARACTER BUILDING.

The mass is composed of its several parts, the body of its associated organs, and the nation of many individuals. All are, in a measure, related to each other and it is absolutely impossible to find conglomerate perfection without perfection in each formative part. The Ego seems so great, the individuality so obtrusive, that it is wellnigh impossible to throw it aside and consider only the common good. Selfishness is innate and merits not reproach so long as its ambition makes for individual improvement; but it is soon seen that self-culture, in its broadest sense, involves more and more of self-obliteration. The highest selfishness thereby becomes the highest unselfishness. There must come denial along the line of all baser motives in order to achieve much in character building. It is essential, however, that a physician should early recognize the importance of those elements that go to the making of a strong and worthy character. The moralist is apt to exhibit intolerance and impatience with those who fall below his standard of conduct, but there is one phase of the question that he does not consider, and that is the dependence of character upon physical conditions and upon varying states of ill health. There is truth in the doctrine that all men cannot build character with the same facility.

Sterling qualities are native to some; they are an inheritance and are observed to be prominent even in childhood. On the other hand, there are many who have a poor heritage and who contend against almost insuperable obstacles in the struggle for character. There is as great a difference in men in this respect as in quality of mind and, indeed, a fine mind makes doubly easy the development of character. There is, in my view, no manner of doubt that physical and nervous defects such as eyestrain, visceroptosis and various forms of neuropathia and psychopathia constitute the underlying fault in many individuals manifesting perversion of character. It is easy to detect gross bodily abnormalities, but we know little, as yet, of the structural, chemical or nutritional changes of the brain cells that may account for many defective moral and mental states. A deep sympathy born of this view of the matter will enable one to withhold harsh and unjust judgment of his fellows, and will also expedite the perception and correction of his own faults.

Bearing in mind this phase of the question, however, the purely psychic side should be given due weight. Right thinking and right acting may be cultivated, determination may be strengthened, the ability to take a larger, broader, finer view of life and its human relations may be made to grow apace by constant and sincere efforts. Much may be done to offset physical drawbacks, as well as inherited nervous and mental defects, by stern striving in the right direction. The influence of environment is of considerable importance, and a change from an unfavorable to a favorable environment will be found to be a signal help. Failure and discouragement will be the lot of many, but if faults and lapses are those of head and not of heart, a saving grace is present still and a higher success will be achieved than will be recognized by him who strives for an ideal. Thus, physicians may help in fostering good citizenship that, in the end, the lawlessness in our beautiful land may, in a measure, be lessened.

The foreseeing critic, who by some may be unjustly called a pessimist, is always unwelcome in our midst, and he who portrays the darker side of our civic, state, and national life is looked upon as a bore. Better a warning pessimism than too long a fatal optimism which is wont to smile while abuse is heaped upon abuse, while fraud in high and low finance robs the public and shatters public confidence, while labor unions and federations violate all the laws of contract; boycott and throttle the public; murder, destroy and pave the way for the reign of anarchy; while half a million men, women and children are annually maimed and killed on the railroads and in the industrial enterprises of the country; while lynching goes grimly on and corruption and political graft thrive; while the law courts allow the vast majority of criminals to go free; while robbery and ugly lawlessness in many forms run riot in the land. I pray you young men and women to promote a high standard of citizenship, so that you may in some degree assist in this mighty national struggle for character that must and will eventually triumph.

There exists a happy belief in the minds of many students of political and social questions that we have in education a remedy for most of the prevalent evils. It is true that education along the right lines offers a practical and scientific means of changing existing conditions, we may hope, in a large measure; and the physician's field for influence in this connection will widen in scope and opportunity when medical inspection and direction of school children is generally established, and when, as is the case in some other countries, the people are represented in the legislature by a larger proportion of physicians than is the case with us at present. There is needed, however, a change in some of the methods of education that more particularly interests physicians, and that has particular reference to the establishment of definite plans for the betterment and uplift of the future generations. All our past and present educational plans have regarded the immediate gain to the individual as greater and more important than the distant, indirect gain to his kind. The chief aim of education has been toward the acquirement of some special knowledge as a means of self-preservation, without regard for the knowledge that man lives in generations and that preservation of the species is of more far-reaching importance. It is not alone desirable that a nation should increase in numbers, but that the individual standard should constantly rise. Education should have charge over the physical as well as the intellectual development of the young to the end that not only mental, but physical disharmonies may be corrected. The best possible strength, form and fitness of body should be the aim of education throughout one generation to another, as only thus is there any hope of emancipating the race from physical degeneracy.

Strong emphasis should be laid by educators upon the prime necessity of well-mating instead of ill-mating. Marriage between defectives should be openly opposed and a proper sense of responsibility in this matter should be firmly implanted. To marry with disease should be prohibited by the strongest arm of effective law. The curb of a higher rationalism should be put upon the deplorably ill-assorted marriages of the present age. Alcoholism, crime, poverty and their wretched accompaniments should disqualify for marriage. Deformity of body or mind should be considered as a ban to marriage. Sentimentalism and emotionalism should be subjected to reason and practical breeding in the human race be brought to a final realization. All these matters come under education and along such lines only may the nation look forward to physical, intellectual and moral betterment. Generation upon generation, century upon century, must roll ere greater standards are reached, but there is no other way. Many must fall and be scattered by the way in order that one may emerge perfect in head and limb, but quality must ever supersede quantity if this human and national problem is to be worked out, and it will not be consummated until educators, one and all, learn that the hereditary influence of protoplasm is extremely far-reaching and powerful, and resists the influence of environment with intense obstinacy.

### CORRELATION OF DISEASES.

As you take up this course, it is well that you should be cautioned not to expect to find diseases standing alone as entities unrelated to other diseased conditions of the body. As you prosecute your studies with observing and analytic minds, you will discover a definite relation between many diseases that may seem, at first glance, wholly independent of each other, as they are wholly unlike each other. There often exists a close causative relation, for instance, between typhoid fever and gallstone disease, the latter being secondary to the former and possibly not appearing until long after recovery from typhoid. The dissimilarity between the symptoms of typhoid fever and those of cholelithiasis is very striking; in the one case the continued temperature, the low pressure, often dicrotic, pulse, the hebetude, the rose rash, the abdominal distension and low delirium with, perhaps, hemorrhagic discharges from the bowels and other symptoms of a pronounced general infection; and in the other case, the sharp attacks of severe epigastric pain with nausea and vomiting appearing in a person with apparent good health, possibly accompanied by some degree of jaundice, with the mahogany colored urine and clay-colored stools incident to that condition. If any temperature supervenes in the course of cholelithiasis, it is usually of an intermittent type. Surely the contrast between these two clinical pictures is apparent to you. Yet the sequence of events in such cases has been found to be this: typhoid bacilli find their way into the gallbladder during the fever and excite a typhoid cholecystitis which persists in a subacute or chronic type until calculi form. The chemical composition of the contents of the gallbladder is altered in inflammation of the organ, and with a nucleus either of calcium bilirubin or clumps of the typhoid bacilli, successive layers of cholesterin are deposited and thus the calculi are built up. Gallstones do not often form unless cholecystitis exists and typhoid fever in this instance is directly responsible for the gallbladder condition, and thus the diseases are correlated.

It has long been observed by physicians that weak heart not infrequently follows typhoid fever, and careful studies of the heart muscle have demonstrated that this weakness is caused by degenerative or inflammatory changes of the myocardium. Various parenchymatous changes occur, but they are not easily differentiated by their clinical manifestations. Albuminous, lardaceous, and fatty degeneration, segmentation of the fibers, nuclear changes, and myocarditis may, one or more, occur. Occasionally there is found an obliterating endarteritis of the smallest arterial twigs of the myocardium and pericardium, as observed by Hayem.

Although the cardiac symptoms dependent upon these vital changes are apt to make themselves manifest during the course of typhoid fever their impress upon the strength of the heart may be felt long after convalescence. If the heart was healthy before the onset of the fever and during its course there occurs sudden pronounced weakness and increased frequency of the pulse, with probable irregularity and arrhythmia, and dilatation of the heart, myocardial involvment is almost certain to have occurred. Cardiac dilatation is at first apt to involve the left side while the right side dilates less frequently and may escape entirely. The apex beat is indistinct and diffuse, or may, in the severest cases, not be demonstrable at all. Upon auscultation, in marked cases, the heart sounds are indistinct and faint, the second pulmonic accentuated and aortic closure enfeebled. Curschmann calls attention to the fact that notwithstanding the alarming nature of the symptoms at times the prognosis is not unfavorable as it is in a similar myocardial condition in diphtheria. I mention these conditions as occurring in the febrile period of typhoid to show you the intimate relation existing between a special infection and one of the most important viscera, but more particularly I wish to call your attention to the later cardiac condition.

Occasionally cases occur in which, after the greatest care and strict rest in bed have been observed, three weeks after defervescence of the fever there appear marked disturbances of the pulse and heart and, perhaps, a mitral insufficiency, relative in character, or due to impaired activity of the papillary muscles.

Fortunately, the prognosis in these cases is favorable so far as immediate danger to life is concerned, but some cases carry cardiac weakness with them for years afterward, and cardiac murmurs are likely to persist in a large percentage of cases that develop them during the fever. Before speaking of the later morbid changes the arteries may undergo because of typhoid fever, let us first consider the acute arterial complications that are sometimes seen during its course. Spontaneous gangrene of the extremities due to arteritis and arterial thrombosis is occasionally seen. From cases presenting this infective arteritis, some French investigators claim to have cultivated typhoid bacilli from the diseased arterial walls. Typhoid gangrene nearly always happens in the lower extremities and, as a rule, only one is involved. Bachmayer, however, described a case of bilateral gangrene of the legs. Keen collected 115 cases of gangrene of the extremities in typhoid fever. In the lower extremities it was present with equal frequency on the right and left sides. It occurred as early as the end of the second week and as late as the seventh week. The gangrene may involve only the toes or may extend half way up the thigh, and in these cases thrombosis is found in the iliac, femoral or posterior tibial arteries.

Although gangrene elsewhere than in the lower extremities occurs with extreme rarity, yet the fingers or the cutaneous covering of the back of the hand have been observed to be its seat. Patry noted gangrene in the distribution of the external carotid. the auricle, the parotid gland and adjacent parts being involved, and Curschmann cites a case of circumscribed cerebromalacia developing right hemiparesis with asphasia in which, post mortem, there were found extensive softening of the middle portion of the left hemisphere and adhesive thrombosis of the artery of the fossa of Sylvius. Welch and Osler also mention cases of typhoid cerebral arterial thrombosis. In the aorta and large arteries internal changes and sclerosis may be observed.

Recent studies have, however, shown that chronic arteriosclerosis may follow typhoid fever, and to Thayer[1] we are indebted for a very interesting investigation of this question. At the outset he considers the influence of acute infections in general upon the production of atheroma of the aorta and arteriosclerosis. The French literature contains many references to this point. Thayer quotes Gilbert and Lion, Crocq and others as having produced fatty sclerotic changes in the aorta of rabbits by slightly injuring the walls and injecting pathogenic bacteria; and Thoma considers typhoid infection an important cause of angiomalacia which he teaches is the primary lesion in arteriosclerotic processes.

In the light of our past views upon the question of the varying degrees of etiologic influence played by different infections in the initiation of arteriosclerosis, it is rather startling to learn

1. Am. Jour. of the Med. Sciences, March, 1904.

the conclusions of Landouzy and Siredey to the effect that "after acute articular rheumatism, typhoid fever appears to give rise to more angiocardiac complications than any of the other infectious diseases." "Among the most important and commonest of these complications are those which arise insidiously during the course or decline of the disease. The angiocardiac lesions are important to recognize, less because of the prognostic reserve which they demand during the course of the disease itself (collapse, sudden death) than for that which they impose upon us for the future." Dr. Thayer succeeded in recalling by means of letters 183 former typhoid fever patients to the Johns Hopkins Hospital, and they were examined as to the condition of their hearts, bloodvessels and blood pressure.

It was found that by comparing 276 healthy individuals and 165 old typhoids, the average blood pressure in the latter was distinctly higher than in the former. Regarding the palpability of the radial arteries, 181 old typhoids were studied with the result that over fifty per cent. of cases about twenty years of age were found to have palpable radials. Irregularity was noted in the distribution of the sclerotic processes in the peripheral vessels. Not uncommonly one radial was found thickened while the other was unaffected, or one temporal artery was tortuous while the other was apparently normal. Two series of old typhoids and healthy individuals were then compared as to the palpability of the radial arteries, and it was found that between the ages of ten and fifty years the old typhoids showed 46.8 per cent. as compared with 17.6 in the normal cases.

An interesting and important part of the study consisted in grouping two lists of cases, old typhoids and normal individuals, none of whom gave a history of severe infectious disease or of alcoholic habits, and it was found that old typhoids gave 45.7 per cent. while non-typhoids showed only 15.1 per cent. of palpable radial arteries.

Investigation of changes taking place in the thyroid gland in typhoid fever has disclosed some interesting facts. Although extremely rare, thyroiditis was nevertheless observed in the European clinics in 40 cases up to 1896. There occurs an acute, painful swelling of a portion of or one-half of the gland; the whole gland is inflamed with great rarity. The inflammation may subside or terminate in suppuration. Very rarely death may occur from suffocation owing to compression or displacement of the trachea, or from abscess rupture into the trachea, but usually nothing serious happens, and recovery is the rule.

This complication is seen more frequently in Switzerland than in other countries, owing to the incidence of goiter which evidently predisposes to it. It has been observed in 19 of 1818 cases of typhoid in Switzerland, while in but five cases in 1376 autopsies in other parts of Europe and the whole series of cases during the great Hamburg epidemic. In some cases typhoid bacilli have been demonstrated to be the cause of the strumitis while other cases are complicated by the presence of pyogenic cocci. The affection appears late in the disease.

Glossitis though uncommonly seen as a complication of typhoid fever yet is present with sufficient frequency to merit mention. I have seen it cause a fatal termination. It usually supervenes during the fastigium, but McCrae reports a case with onset the twenty-fifth day of normal temperature. It was associated with a typical relapse, and recovery was the outcome. In typhoid glossitis the tongue becomes enormously swollen and dark red, in fatal cases almost black. It may assume proportions causing it to protrude and fill up the pharynx causing death by suffocation and exhaustion. Dysphagia is an early symptom and soon the patient may be wholly unable to swallow even water. This complication is most horrible and agonizing in fatal instances. Suppuration may occur and rupture of the abscess take place into the pharynx or the larynx. In some instances the neck becomes enormously swollen. the eyes bloodshot and protruding and the face swollen and cyanotic. The tongue soon becomes dark coated and the breath is apt to have a putrid odor. Gangrene of the tongue may occur.

Considerable importance attaches to the question of ulceration of the mucous membrane of the larynx and perichrondritis in typhoid fever. Infiltration of lymph follicles has been found upon the posterior wall of the larnyx and upon the base of the epiglottis, and typhoid bacilli have been demonstrated in these follicles. Like the lymph follicles in the intestine, these tend to ulceration and necrosis. Ulceration of the larynx may, however, occur in any other way in typhoid fever. Incident to the commonly associated laryngitis with cough there may occur small erosions upon the wall of the larynx between the arytenoid cartilages and the attachment of the vocal bands, and these erosions may extend during the period of greatest tissue depression in the disease, and become more or less extensive ulcerations. The situation of the ulceration is always on the posterior wall, but there seems to be insufficient warrant for regarding it as decubital. If secondary perichondritis and necrosis of cartilage take place, the cricoid and arytenoid cartilages are usually involved.

Turning now from typhoid fever to a consideration of a few other diseases accompanied by important complications and followed by momentous sequelæ, let me call your attention first to acute infectious polyarthritis. This disease may often be traced to some antecedent affection as, for instance, follicular or parenchymatous tonsillitis which affords the inoculation of the body with streptococci, staphylococci, or possibly with some undiscovered microörganism that is the specific cause of the disease. Given the establishment of acute multiple arthritis of this type, endocarditis occurs secondarily in a large proportion of cases. Endocarditis in its turn frequently causes valvular disease which is later to blame for hypertrophy and dilatation of the heart. In time the cycle of disease induces pulmonary congestion, and soon there develops passive congestion of the liver, gastrointestinal tract and kidneys. Thus, a cyanotic kidney is related, as between cause and effect, to the follicular tonsillitis. The same sequence of events often follows scarlet fever as it is prone to excite endocarditis. Scarlet fever also has a special predisposition to excite a more or less acute parenchymatous nephritis. In some cases this assumes the form of a glomerulonephritis and with total suppression of urine the victim of the disease may promptly die of uremia. Sometimes a more chronic form of nephritis develops and secondary changes in the circulatory apparatus generally supervene.

So, also, a special localised infection that may be accounted trivial, may prove deadly through the highways and byways of the system. Thus a small boil may turn out to be the seat of inoculation of some virulent organism and general septicemia results. Or gonorrhea may cause ulcerative endocarditis with its widespread septic and embolic effects and fatal termination. Or septic endometritis, excited by careless artificial abortion, may cause septic thrombophlebitis in a uterine vein, a fragment of the intravenous thrombus may be swept off and carried through the veins to the right ventricle and from thence be forced to a pulmonary arterial branch which it plugs, thereby forming a septic infarct in the lung and a small abscess near the periphery of the lung which may rupture into the pleural cavity and establish a fatal pneumothorax. This cycle of events was observed by several of us in a case in the Buffalo General Hospital some years ago, illustrating in a tragic manner the correlation of disease processes.

In closing let me wish you good health, pleasure and success in your college life. May goodfellowship abound and may you squarely face your tasks and conquer them in a spirit of undaunted cheer, willingness and courage!

436 FRANKLIN STREET.

---

"BUT," asked the young doctor, "why do you always order champagne for every new patient that comes to you?

"Because, my boy," replied the old practitioner, "I can judge by what the patient says whether or not he can afford it. That helps me when I come to make out my bill."—*Philadelphia Press.*

